# Fever of unknown origin in China: evaluation of 918 cases during a ten-year-period of study

**DOI:** 10.1186/1471-227X-12-S1-A2

**Published:** 2012-12-18

**Authors:** Qing-yi Meng, Jing-ling Ma

**Affiliations:** 1 Emergency Department, Chinese PLA General Hospital, Beijing, China

## Background

Fever of unknown origin (FUO) is a challenging problem worldwide. FUO, as defined by Petersdorf and Beeson in 1961 [1] , includes illness persisting for 3 weeks or more, occasional episodes of a fever 38.3°C or more, and an unclear cause upon examination after one week of hospitalization. Recently, only one retrospective study of FUO have been conducted in the south China, but there has been no good data about FUO in the north China, especially in the top-level general hospital. Based on these considerations, a 10-year retrospective study among patients with FUO in a 3100-bed teaching hospital in northern China was conducted to explore the geographic profile of FUO.

## Method

We conducted a retrospective chart review of all patients admitted to the Chinese general hospital, Beijing, China, between January 2001 and December 2010 who were diagnosed as having FUO in their admission and/or discharge diagnoses. There were 918 patients with prolonged fever fulfilling the Petersdorf and Beeson of FUO criteria. During the ten-year period, 1,002,828 patients were hospitalized, and percentage of FUO was 0.09% (918/1,002,828) in our hospital.

## Results

During the ten-year period of the study, 918 patients were followed up as FUO. The mean age was 49 years, ranging from 13–82 years. Males accounted for 521 (56.8%) of the sample, with a male: female ratio of 1:076. The mean duration of hospitalization was approximately 27.6 days, ranging from 9–92 days. The final diagnoses are presented in Table 1.

**Table 1:** Causes of fever unknown origin.

Diagnostic category	number of patients (n =918)	%
Infection	358	38.9
Collagen vascular disease	298	32.5
Neoplasm	120	13.1
Miscellaneous	82	8.9
No diagnosis	60	6.5

## Conclusion

Our findings suggest that the spectrum of diseases causing FUO in the Chinese people is characteristic. The infections were the predominant cause of FUO (38.9%) in the current study, which was lower with the findings of previous studies (49.4% -52.6%). Collagen vascular disease and neoplasm also was the more common cause of FUO. And the miscellaneous diseases and undiagnosed FUO accounted for 8.9% and 6.5% of the samples separately.

## References

[B1] Petersdorf RG Beeson PB Fever of unexplained origin: report on 100 cases. Medicine (Baltimore) 1961 1 30 13734791 10.1097/00005792-196102000-00001

